# Sympathetic nerve inhibition enhances calvarial bone repair via senescent macrophage-induced osteogenesis and angiogenesis

**DOI:** 10.1038/s41420-025-02886-y

**Published:** 2025-12-10

**Authors:** Lei Zhao, Zhaoning Xu, Peiru Zhao, Zhiying Pang, Yu You, Chao Wu, Di Zhu, Meiling Su, Ning Zhang, Jian Luo, Yiyun Wang

**Affiliations:** 1 Yangzhi Rehabilitation Hospital (Shanghai Sunshine Rehabilitation Center), Tongji University School of Medicine, Shanghai, PR China; 2 Shanghai East Hospital, School of Medicine, Tongji University, Shanghai, PR China; 3 Musculoskeletal Research Laboratory, Department of Orthopaedics & Traumatology, The Chinese University of Hong Kong, Hong Kong SAR, PR China

**Keywords:** Experimental models of disease, Stem-cell research

## Abstract

Bone regeneration is a tightly coordinated process involving multiple cellular and molecular components, with emerging evidence highlighting the pivotal role of the nervous system, especially the sympathetic nervous system, in modulating skeletal repair. However, the mechanistic details of neuro-skeletal interactions during bone healing remain elusive. Here, we inhibited peripheral sympathetic nerves using 6-hydroxydopamine (6-OHDA) in a murine calvarial defect model and performed single-cell RNA sequencing on the injury sites at 7 and 14 days post-injury to delineate the cellular landscape underlying regeneration. Our analyses revealed activation of neurogenesis-associated pathways and dynamic crosstalk between neural and skeletal cells following injury. Sympathetic nerve inhibition significantly enhanced calvarial bone repair, characterized by downregulation of *Capn6* in suture mesenchymal cells, increased formation of H-type blood vessels, and the emergence of a distinct macrophage subset exhibiting senescence-associated phenotypes. Importantly, pharmacological clearance of senescent cells by senolytic agents abrogated the regenerative benefits conferred by sympathetic blockade. Mechanistically, sympathetic inhibition promoted angiogenesis and osteogenesis by facilitating interactions between suture mesenchymal cells and endothelial cells, while the senescent-like macrophages contributed to bone repair via secretion of osteogenic cytokines. Collectively, these findings uncover a critical role of sympathetic nerves in regulating the bone healing niche and identify potential therapeutic targets to enhance skeletal regeneration. These insights may pave the way for the development of neuromodulatory or senescence-targeted therapies to promote bone repair in challenging clinical scenarios such as cranial bone defects, non-union fractures, or aging-associated impaired healing.

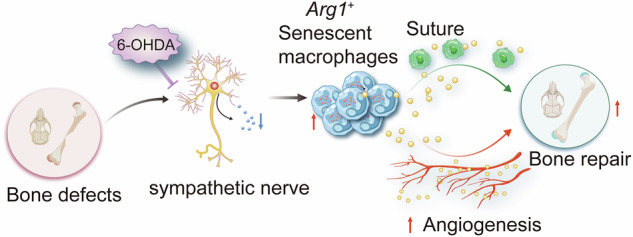

## Introduction

Emerging evidence has revealed the crucial role of the nervous system, particularly the sympathetic nervous system, in regulating tissue repair and regeneration [1]. Sympathetic nerves, which densely innervate bone tissue, influence bone metabolism and repair through adrenergic signaling [2]. This regulation occurs via β-adrenergic receptors on osteoblasts, osteoclasts, and endothelial cells, modulating osteogenesis, angiogenesis, and inflammation [3–5]. Sympathetic nerve activity typically increases in response to defect, triggering stress responses that can either enhance or impair tissue repair depending on the context [6]. Sympathetic nerve inhibition reduces local inflammation and promotes the recruitment and differentiation of osteoprogenitor cells [7]. Additionally, the modulation of immune cell phenotypes by sympathetic nerves is critical for the repair process, as the neuroimmune crosstalk regulates macrophage polarization and inflammatory resolution [8, 9].

A deeper understanding of how sympathetic innervation shapes the cellular microenvironment during repair could uncover novel therapeutic strategies for enhancing tissue regeneration across various contexts. Many works have illuminated the critical roles of mesenchymal stem cells [10, 11], immune cells [12], and vascular components [13] in orchestrating the repair process. However, the sympathetic nervous system regulates interactions between cells, the overall cellular changes, and cell-to-cell communication following its activation or inhibition is ambiguous. How sympathetic nervous system influence cells function and subsequently affect bone defect repair requires further investigation.

Using a mouse calvarial defect model, we employed scRNA-seq to construct a comprehensive cellular atlas of the repair process, integrating data from both uninjured controls and injured samples across multiple time points. Our analysis revealed that sympathetic nerve inhibition enhances calvarial bone repair by modulating mesenchymal differentiation, macrophage dynamics, and H-type vessel formation. Notably, it induced a senescent macrophage phenotype that promoted regeneration via osteogenic and angiogenic factors. These findings underscore the therapeutic potential of modulating peripheral sympathetic nerve activity and targeting specific cellular subpopulations, including senescent cells, to optimize bone regeneration.

## Result

### Peripheral sympathetic nerve inhibition promotes calvarial defect repair

To systematically characterize the cellular and molecular changes during calvarial bone repair, we analyzed single-cell RNA sequencing data from day 0, day 7, and day 28 post-injury (GSE245094) [14]. Subsequently, differential expression gene (DEG) analysis was performed by comparing single-cell data from injured mice at these two time points with uninjured controls (Supplementary Fig. S1A, Supplementary Table S1). Importantly, a substantial proportion of the secretory proteins consistently upregulated during calvarial defect healing were enriched in biological processes associated with neuroregeneration and growth (Supplementary Fig. S1B), suggesting that neuroregulatory mechanisms play a pivotal role in the repair process following calvarial bone defect. Furthermore, intercellular communication by CellChat showed that PTN, SPP1 and TNF pathways were the highest communication probability in defect samples (Supplementary Fig. S1C). It has been reported that PTN is a neurotrophic factor for spinal motor neurons [15]. Peripheral nerves are involved in various physiological and pathological processes in bone, and changes in these nerves following a bone fracture are critical for initiating bone regeneration [16]. Additionally, activation of the sympathetic nervous system has been shown to have a direct association with bone loss [16, 17].

**Fig. 1:**
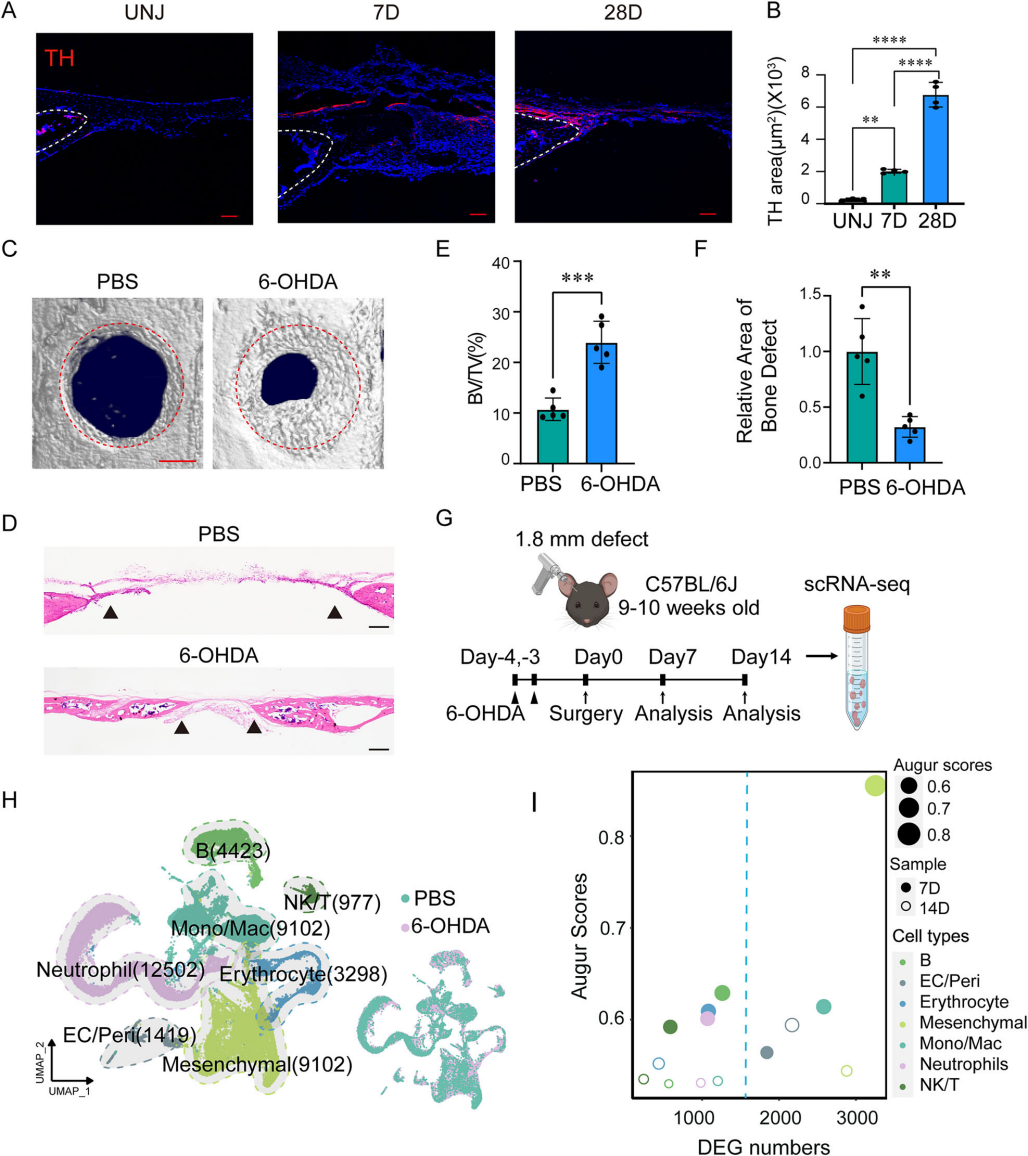
Sympathetic nerve inhibition promotes calvarial defect repair and cellular disturbances. **A** Immunohistochemical staining of TH^+^ (Tyrosine hydroxylase) sympathetic nerve fibers at the same edge from none defect, calvarial bone defect at day 7 post-defect and day 28 (28D) post-defect, appearing red, and **B** measurement of red immunoreactivity in the skull defect area. DAPI counterstain appears blue in all images. Bone edges are marked with dashed white lines. In graphs, each dot represents a single animal; *n* = 4 per group. Data are represented as mean ± SD. Scale bar represents 100 μm. **C** Micro-CT reconstructions of the defect site in a top-down view among animals treated with PBS or 6-OHDA. Analysis performed at day 28 post-defect. Margins of original defect are indicated by dashed red lines. Scale bar, 500 μm. **D** H&E stain of representative coronal cross-section of the healing defect site from PBS or 6-OHDA treatment mice. Scale bar, 250 μm. Micro-CT quantification of bone healing, among PBS or 6-OHDA treatment mice, including **E** bone volume/tissue volume (BV/TV) and **F** relative area of bone defect. **G** The workflow of single-cell RNA sequencing (Created in BioRender. Bian, Z. (2025) https://BioRender.com/t10k902). **H** The UMAP plot displaying different cell types identified by scRNA-seq (left). The colors represent cell cluster compartments under PBS and 6-OHDA treatments (right). **I** Bubble plot illustrating the number of DEGs and Augur scores across cell types in PBS and 6-OHDA-treated samples. Dot size represents the Augur score, solid dots correspond to samples at day 7 post-defect, and hollow dots represent samples at day 14 post-defect. Different colors indicate different cell subpopulations.

To further explore whether sympathetic nervous changes occur after bone defect, we performed tyrosine hydroxylase (TH) staining on calvarial bone samples collected on day 7 and 28 post-defect and found a significant increase in sympathetic nerve innervation following the calvarial bone defect (Fig. 1A, B). Local injections of 6-hydroxydopamine (6-OHDA) were administered to inhibit peripheral sympathetic nerves surrounding the calvarial bone (Supplementary Fig. S1D, E). Next, frontal bone healing was assessed following sympathetic nerves inhibition (6-OHDA) or non-inhibition (PBS) over 28-day period. MicroCT reconstructions and cross-sectional images demonstrated enhanced bone formation in 6-OHDA-treated mice compared to PBS-treated mice (Fig. 1C). Hematoxylin and eosin (H&E) staining confirmed notable improvement in healing between bony fronts in 6-OHDA treated mice (Fig. 1D, black arrowheads). Quantitative micro-CT metrics of bone healing showed significant improvements in 6-OHDA treated mice, including a 123% increase in fractional bone volume (BV/TV; Fig. 1E) and a 60% reduction in the mean diameter of the bone defect area (Fig. 1F).

To further investigate how sympathetic nerve inhibition promotes calvarial bone defect repair, single-cell RNA sequencing was performed on calvarial bone samples collected 7 and 14 days after frontal bone defect induction with sympathetic nerve inhibition (Fig. 1G). After data pre-processing and quality control, transcriptomic data were obtained for 40903 cells. Using unsupervised graph clustering, these cells were partitioned into 8 subsets, including Mesenchymal cells (9102), Erythrocytes (3298), Neutrophils (12502), Monocytes/Macrophages (Mono/Mac, 9102), B cells (4423), Endothelial cells/Pericytes (EC/Peri, 1419), and NK/T cells (977) clusters (Fig. 1H), visualized by uniform manifold approximation and projection (UMAP) and labeled according to marker gene expression (Supplementary Fig. S1F). Inhibiting sympathetic nerve function did not result in significant changes in cell types (Fig. 1H). Furthermore, we identified 116 genes that were significantly up-regulated at both 7 and 14 days post-defect at sympathetic nerve inhibition group compared to control group (Supplementary Fig. S1G, Supplementary Table S2). KEGG pathway enrichment analysis revealed the MAPK signaling pathway, FoxO signaling pathway and TNF signaling pathway were enriched in sympathetic nerve inhibition group (Supplementary Fig. S1H). We subsequently utilized the Augur [18] software to analyze the cellular disturbances induced by sympathetic nerve inhibition. At 7d post-defect, the most significant disturbance was observed in mesenchymal cells, while at 14d post-defect, endothelial cells/pericytes and monocytes/macrophages exhibited the greatest disturbances (Fig. 1I). These results suggest that inhibition of the sympathetic nervous system has a marked impact on mesenchymal cells, monocytes/macrophages and endothelial/pericytes cells, thereby promoting the repair of calvarial bone defects.

### Suppression of *Capn6* expression promotes osteogenesis in the suture following sympathetic nerve inhibition

**Fig. 2:**
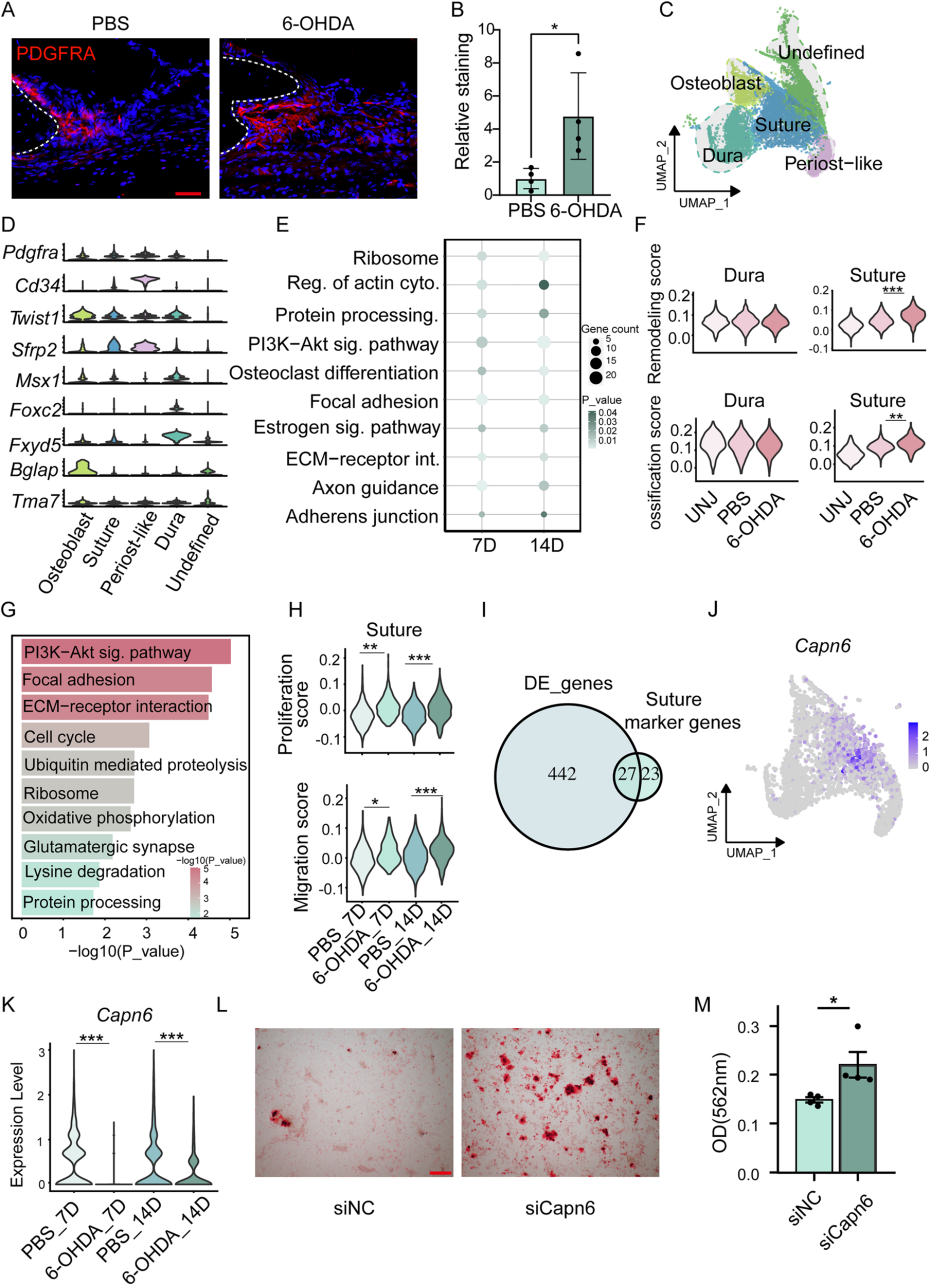
Single-cell sequencing analysis reveals the dynamic changes in different subpopulations of mesenchymal cells after sympathetic nerve inhibition. **A** Immunohistochemical staining of PDGFRA at the defect edge from PBS or 6-OHDA treatment mice. **B** Quantification of PDGFRA immunoreactivity within the calvarial defect. Dashed white lines indicate bone edge. Scale bar, 100 μm. DAPI counterstain appears blue in all images. In graphs, each dot represents a single animal; *n* = 4 per group. The fluorescence area was quantified using ImageJ software, and the values were normalized to the PBS control group, which was set to 1. Statistical comparisons between two groups were performed using an unpaired two-tailed Student’s t-test. Data are presented as mean ± SD. **C** UMAP plot illustrating the further clustering results of mesenchymal cells. **D** Violin plots showing the expression of marker genes across different mesenchymal subpopulations. **E** Bubble plot highlighting signaling pathways in mesenchymal subpopulations at day 7 and day 14 post-defect following 6-OHDA treatment. **F** Violin plots presenting tissue remodeling and osteogenesis gene set scores of dura and suture tissues under different treatment conditions. **G** Signaling pathways enriched in upregulated genes in the suture following sympathetic nerve inhibition. **H** Violin plots illustrating proliferation scores and migration score of suture under different treatment conditions. **I** The intersection of upregulated differentially expressed genes in the suture after sympathetic nerve inhibition and suture-specific marker genes. **J** DimPlot shows the expression of *Capn6*. **K** Violin plots illustrating *Capn6* expression across different treatment groups. **L** Alizarin Red staining demonstrates that inhibiting *Capn6* expression in the suture enhances its osteogenic potential. **M** OD values measured after *Capn6* inhibition indicate enhanced mineralization.

Stem cells play a crucial role in calvarial bone defect repair [19]. Immunofluorescence staining revealed a marked increase in mesenchymal stem cell markers PDGFRA and osteogenic marker COL1A1 following sympathetic nerve inhibition (Fig. 2A, B, Supplementary Fig. S2A, B). Additionally, the proliferation and osteogenic scores of the mesenchymal stem cell subpopulation were significantly enhanced following sympathetic nerve inhibition (Supplementary Fig. S2C, D). Previous studies have shown that stem/progenitor cells from the suture, dura, and periosteum have been reported to contribute to calvarial repair [14, 20–22]. To further investigate this, we performed a focused analysis of the mesenchymal cell cluster, identifying five subclusters: osteoblasts (expressing *Bglap*), suture (expressing *Sfrp2*), dura (expressing *Foxc2* and *Fxyd5*), periost-like (expressing *Pdgfra* and *Cd34*), and an undefined cell type (expressing *Tma7*), based on data collected on day 7 and day 14 after bone defect induction following sympathetic nerve inhibition (Fig. 2C, D). Subsequently, we focused primarily on osteoblasts, suture, dura, and periost-like cell types. DEGs were significantly enriched in osteogenesis-related pathways, including the PI3K-Akt signaling pathways, osteoclast differentiation, and focal adhesion (Fig. 2E). Stemness analysis indicated that the suture subpopulation possessed the highest potential for stem cell differentiation (Supplementary Fig. S2G), while dura exhibited the highest tissue remodeling and osteogenic scores (Supplementary Fig. S2E, F). However, following defect, the tissue remodeling and osteogenic scores of dura did not show significant changes. In contrast, the scores for suture showed a significant increase post-defect (Fig. 2F). Moreover, in the day 14 group with sympathetic nerve inhibition (6-OHDA_14D), the osteogenic and tissue remodeling scores for suture were significantly higher than those in the uninjured group (Fig. 2F), suggesting that sympathetic nerve inhibition enhances the osteogenic and tissue remodeling functions of suture. Further pathway analysis revealed that the suture subpopulation following sympathetic denervation was highly enriched in PI3K-Akt signaling, focal adhesion, and ECM–receptor interaction (Fig. 2G). In addition, sympathetic nerve inhibition significantly increased the proliferation and migration scores of suture (Fig. 2H). To investigate how sympathetic inhibition regulates the osteogenic potential of the suture, we intersected the differentially expressed genes with suture-specific genes and identified *Capn6* as both significantly downregulated following sympathetic blockade and specifically enriched in the suture (Fig. 2I–K, Supplementary Fig. S2H). Functional validation using gene knockdown experiments demonstrated that suppression of *Capn6* expression in the suture effectively enhances its osteogenic capacity (Fig. 2L, M, Supplementary Fig. S2I–K). Thus, these findings identify *Capn6* as a key negative regulator of suture osteogenesis and highlight the suture as a critical target of sympathetic nerve modulation during calvarial bone repair.

### Endothelial cell sub clustering reveals an increase in type H vessels following sympathetic nerve inhibition

**Fig. 3:**
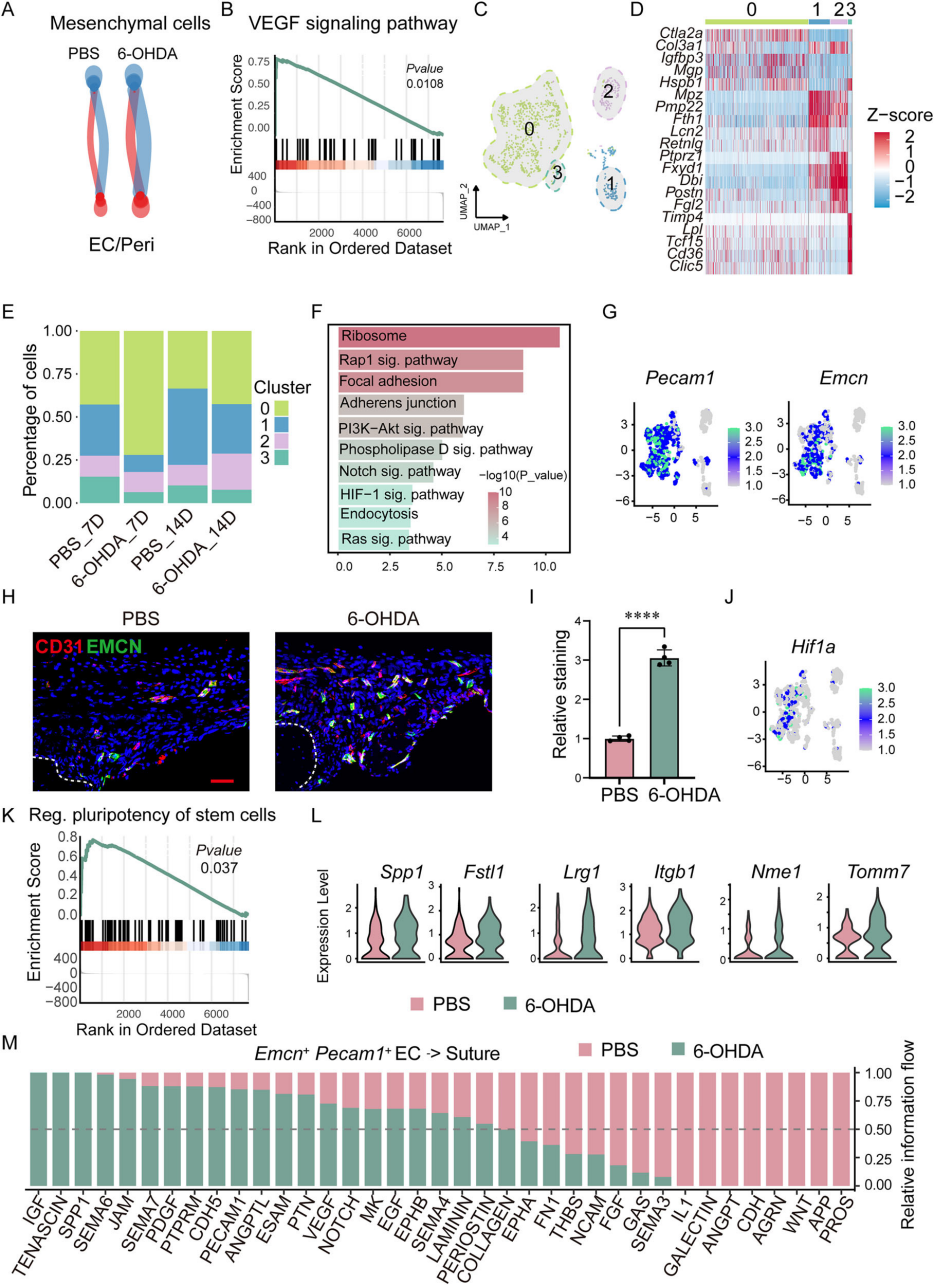
Inhibition of sympathetic nerves enhances the formation of H-type vessels. **A** Changes in cell–cell communication between endothelial cells/pericytes and mesenchymal cells in mice treated with PBS or 6-OHDA. **B** GSEA analysis of the VEGF signaling pathway in endothelial cells/pericytes after 6-OHDA treatment. **C** Uniform Manifold Approximation and Projection (UMAP) plot illustrating the further clustering of endothelial cell subpopulations. **D** Heatmap displaying markers specifically expressed in subpopulations of endothelial cells. **E** Changes in the proportions of different endothelial cell subpopulations under various treatment conditions. **F** Barplot shows the signaling pathways enriched among genes specifically expressed in the C0 subcluster. **G** Expression levels of *Pecam1* and *Emcn* across different endothelial cell subpopulations in 6-OHDA treatment at day 28. **H** Immunohistochemical staining of CD31^+^ EMCN^+^ at the defect edge from PBS or 6-OHDA treatment mice, appearing CD31 (red) EMCN (green), and **I** quantification of CD31^+^ EMCN^+^ immunoreactivity within the calvarial defect. Dashed white lines indicate bone edge. Scale bar, 70 μm. DAPI counterstain appears blue in all images. In graphs, each dot represents a single animal; *n* = 4 per group. Data are represented as mean ± SD. **J** DimPlot shows the expression of *Hif1a*. **K** GSEA analysis of the regulation pluripotency of stem cells pathway in *Emcn*^+^ *Pecam1*^+^ endothelial cells after 6-OHDA treatment. **L** Expression levels of *Spp1*, *Fstl1*, *Lrg1*, *Itgb1*, *Nme1*, and *Tomm7* in *Emcn*⁺ *Pecam1*⁺ endothelial cells under PBS and 6-OHDA treatment conditions. **M** Significantly altered signaling pathways in cell–cell communication between *Emcn*^+^ *Pecam1*^+^ endothelial cells and suture cells at PBS versus 6-OHDA treatment mice.

Through cell-cell communication analysis, we observed a significant increase in the interactions between endothelial cells/pericytes and mesenchymal cells (Fig. 3A). Endothelial cells play a critical role in bone defect repair [23]. Previous studies have reported that VEGF promotes osteogenic differentiation of bone progenitor cells [24]. In our study, we found that following sympathetic nerve inhibition, the VEGF signaling pathway in the endothelial cell/ pericytes subpopulation was significantly enriched (Fig. 3B). Much attention has been paid to endothelial subtypes involved in osteo-angiogenic coupling, including type H vessels characterized by high expression of endomucin (*Emcn*) and *Pecam1*. To further investigate endothelial cell heterogeneity, we performed further clustering analysis, identifying four distinct subpopulations of endothelial cells (Fig. 3C, D). Notably, there was a significant increase in the proportion of the C0 subpopulation in the sympathetic nerve inhibition group at both day 7 and day 14 after bone defect group (6-OHDA_7D, 6-OHDA_14D, Fig. 3E). GO enrichment analysis revealed that the markers specifically highly expressed in the C0 cell subpopulation were mainly enriched in pathways related to the ribosome, PI3K-Akt, and HIF-1 signaling (Fig. 3F). Further analysis of the C0 subpopulation revealed high expression levels of *Pecam1* and *Emcn* (Fig. 3G). To further examine this, immunofluorescent staining for Pecam1 and Emcn was performed (Fig. 3H, I). Results showed a progressive increase in Pecam1^high^ Emcn^high^ vessels in 6-OHDA group (Fig. 3H, I). Moreover, the *Pecam1*^high^ *Emcn*^high^ (C0) subcluster demonstrated higher expression of *Hif1α* (Fig. 3J, Hypoxia inducible factor 1 subunit alpha) - high *Hif1α* expression has been previously linked to a type H vessel phenotype. Together, these data suggest an increase of endothelial cells with a type H phenotype after 6-OHDA treatment. Additionally, the *Pecam1*^high^ *Emcn*^high^ (C0) subcluster showed significant enrichment of signaling pathways that regulate stem‑cell pluripotency (Fig. 3K). We also identified numerous secreted‑protein genes—such as *Spp1*, *Fstl1*, *Lrg1*, *Itgb1*, *Nme1*, and *Tomm7* (Fig. 3L)—among the genes up‑regulated in this subcluster after sympathetic nerve inhibition. High expression of these secreted proteins has been reported to promote both osteogenic differentiation of stem cells and angiogenesis, suggesting that the *Pecam1*^high^ *Emcn*^high^ subcluster releases cytokines that boost stem‑cell activity and thereby accelerate defect repair. To further examine how sympathetic nerve inhibition influences endothelial cell–suture crosstalk, we analyzed cell-cell communication between these populations in 7‑day post‑defect samples treated with 6‑OHDA. Ligand‑receptor signaling patterns revealed a marked increase in osteogenesis‑related pathways—including CD34, IGF, and PDGF signaling—which remained consistently elevated in the 6‑OHDA group (Fig. 3M). Collectively, these findings demonstrate that sympathetic nerve inhibition facilitates bone defect repair by promoting type H vessel formation and potentiating stem cell–driven osteogenesis through endothelial cell-mediated signaling.

### Sympathetic nerve inhibition promotes the generation of senescent cells, thereby facilitating calvarial bone repair

**Fig. 4:**
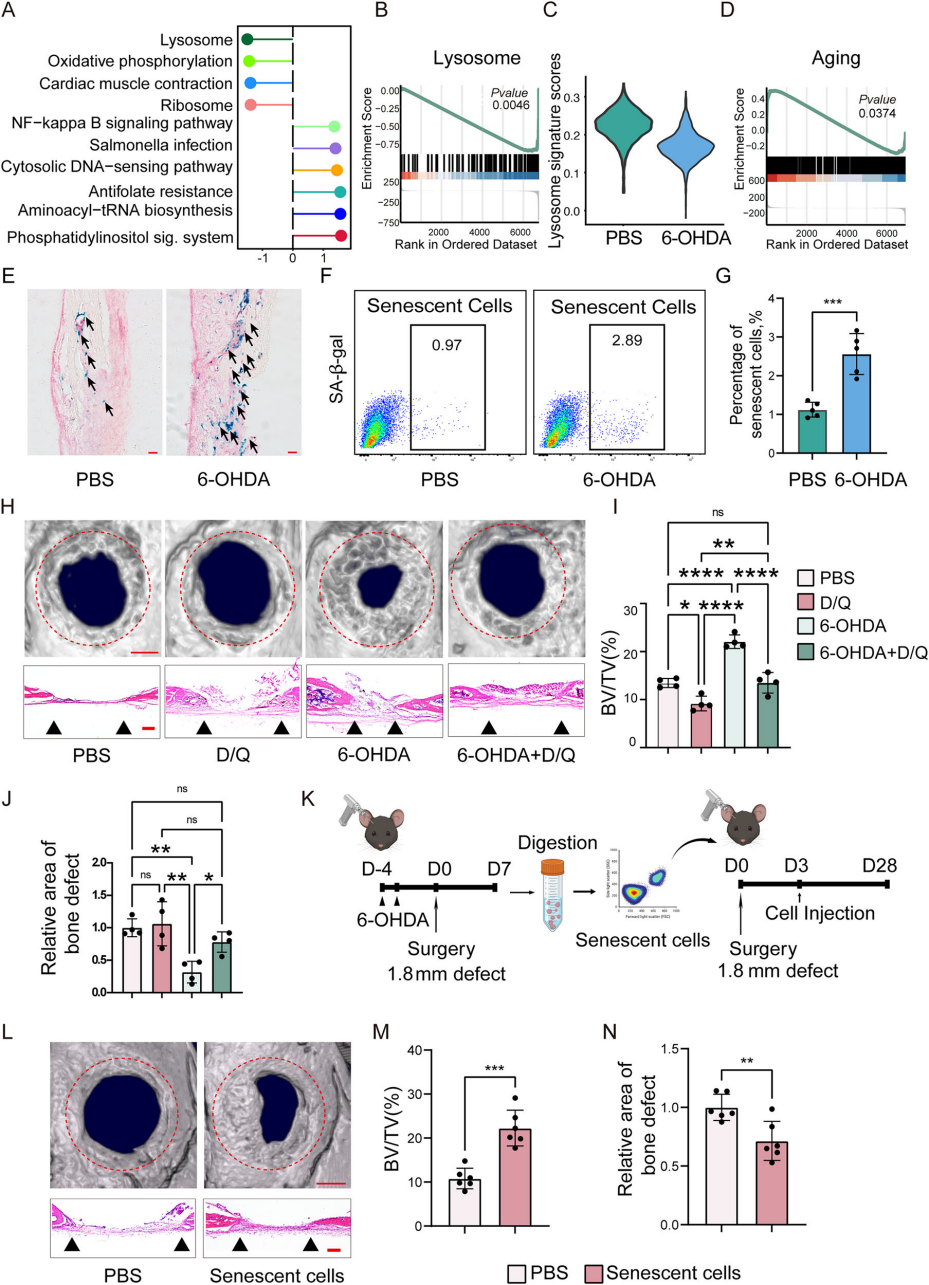
Senolytics counteracts the accelerated cavarial defect healing induced by sympathetic nerve inhibition. **A** Lollipop plot showing signaling pathways in monocytes/macrophages in both the 6-OHDA treatment group and the PBS treatment group at day 14 post-defect. **B** GSEA analysis revealing the enrichment of lysosome signaling pathway in monocytes/macrophages in the 6-OHDA treatment group at day 14 post-defect. **C** Violin plot showing the lysosome gene set scores in monocytes/macrophages in both the 6-OHDA treatment group and the PBS treatment group at day 14 post-defect. **D** GSEA analysis revealing the enrichment of aging pathway in monocytes/macrophages in the 6-OHDA treatment group at day 14 post-defect. **E** SA-β-gal staining of calvarial bone from PBS or 6-OHDA treatment mice. Scale bar, 50 μm. **F** Flow cytometry showing the percentage of senescent cells from PBS or 6-OHDA treatment mice. **G** Quantification of senescent cells by flow cytometry in PBS- and 6-OHDA-treated groups. **H** Micro-CT reconstructions of the defect site in a top-down view and H&E stain of representative coronal cross-section of the healing defect site among animals treated with PBS, D/Q, 6-OHDA or 6-OHDA + D/Q, (D/Q indicates senolytics Dasatinib and Quercetin). Analysis performed at day 28 post-defect. Margins of original defect are indicated by dashed red lines. Scale bar, 500 μm. Micro-CT quantification of bone healing, among PBS, D/Q, 6-OHDA and 6-OHDA + D/Q treatment mice, including **I** bone volume/tissue volume (BV/TV) and **J** relative area of bone defect. **K** Workflow illustrating the process of senescent cell sorting and transplantation (Created in BioRender. Bian, Z. (2025) https://BioRender.com/t10k902). **L** Micro-CT reconstructions of the defect site in a top-down view and H&E stain of representative coronal cross-section of the healing defect site among animals treated with PBS or senescent cells. Analysis performed at day 28 post-defect. Margins of original defect are indicated by dashed red lines. Scale bar, 500 μm. Micro-CT quantification of bone healing, among PBS and senescent cells treatment mice, including **M** bone volume/tissue volume (BV/TV) and **N** relative area of bone defect.

Augur analysis identified macrophages as one of the most responsive cell types following sympathetic nerve inhibition (Fig. 1I), consistent with previous reports that sympathetic nerves regulate macrophage activity [25]. To further investigate, we analyzed macrophage subpopulations and found significant downregulation of lysosome-related pathways at day 14 post-defect in the 6-OHDA group, as revealed by GSEA and reduced lysosome gene set scores (Fig. 4A–C). Lysosomal dysfunction, a hallmark of cellular senescence, leads to metabolite accumulation and is closely linked to aging processes [26, 27]. Additionally, pathway associated to oxidative phosphorylation was significantly enriched (Fig. 4A), which are strongly implicated in cellular senescence [28, 29]. Using a literature-derived senescence gene set, we found increased enrichment scores in macrophages from the 6-OHDA group (Fig. 4D). β-galactosidase staining of calvarial bone sections confirmed higher senescence levels in sympathetic nerve–inhibited mice (Fig. 4E), which was further supported by flow cytometric analysis of β-gal–positive cells (Fig. 4F, G). To explore the relationship between the accumulation of senescent-like cells and calvarial defect repair, we treated mice with senolytics Dasatinib and Quercetin (D/Q) before inducing sympathetic nerve inhibition and calvarial defect. This treatment abrogated the defect repair effects of sympathetic nerve inhibition (Fig. 4H–J). To further validate that sympathetic nerve inhibition promotes the generation of senescent cells and thereby facilitates calvarial bone defect repair, we isolated senescent cells from the injury site of mice seven days after 6-OHDA treatment and calvarial bone defect. These senescent cells were then transplanted into the calvarial defect sites of receptor mice (Fig. 4K). At 28 days post-transplantation, bone repair was assessed. Micro-CT reconstructions and cross-sectional images revealed enhanced bone formation in mice receiving senescent cells compared to PBS-treated controls (Fig. 4L). Hematoxylin and eosin (H&E) staining further confirmed improved healing between the bony fronts in the 6-OHDA-treated group (Fig. 4L, black arrowheads). Quantitative micro-CT analysis demonstrated significant improvements in bone regeneration in the senescent cell–treated group, including a 106% increase in bone volume fraction (BV/TV; Fig. 4M) and a 40% reduction in the mean diameter of the bone defect area (Fig. 4N).

### Sympathetic nerve inhibition promotes the generation of *Arg1*^+^ macrophages with senescent features, thereby facilitating calvarial bone repair

**Fig. 5:**
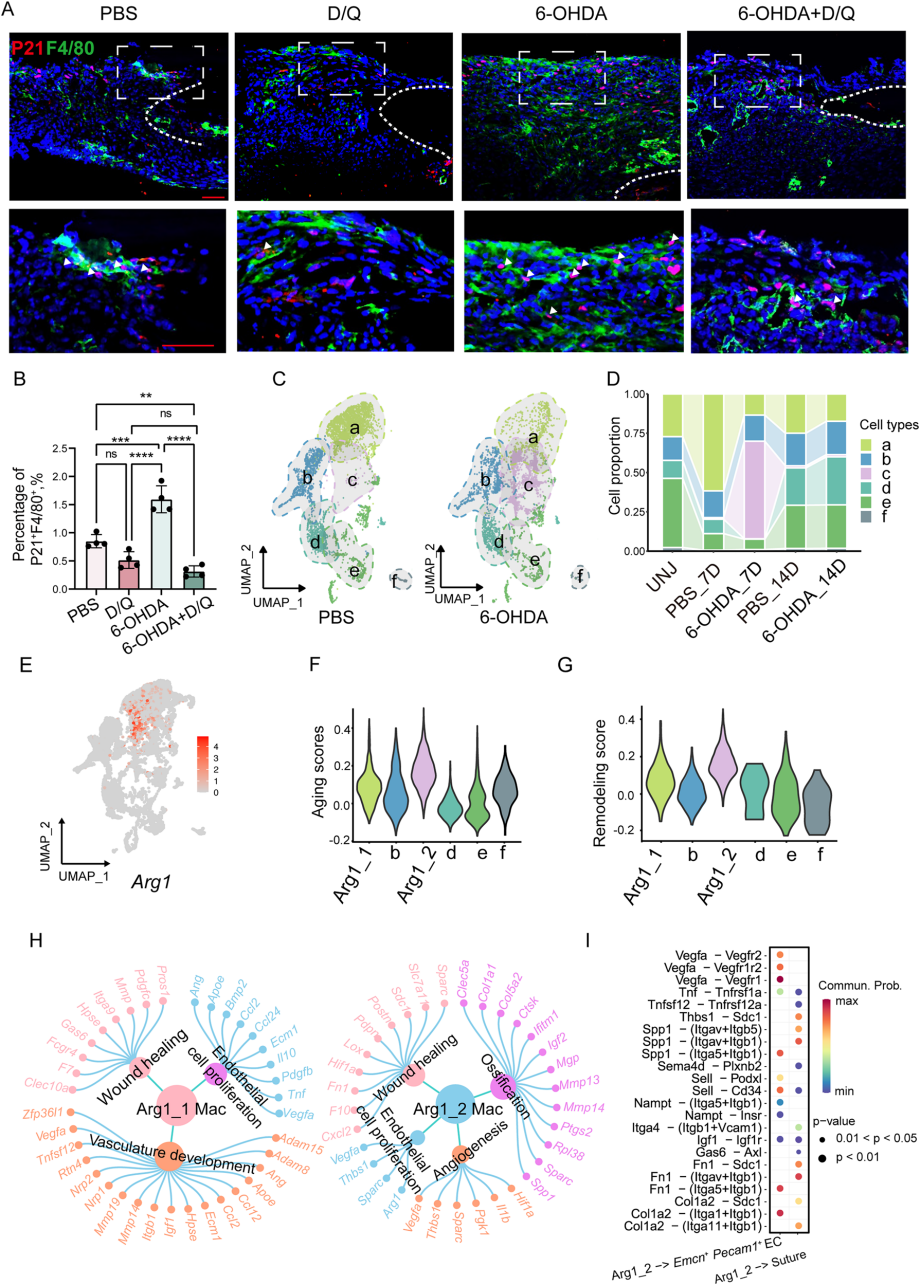
Sympathetic Nerve Inhibition Induces Senescent Macrophage Subpopulation to Promote Calvarial Defect Repair. **A** Immunohistochemical staining of P21^+^ F4/80^+^ cells at the defect edge from PBS, D/Q, 6-OHDA and 6-OHDA + D/Q treatment mice Green represents F4/80, while red represents P21. If the nucleus appears red and the cell membrane appears green, the cell is considered double-positive. Dashed white lines indicate bone edge. White arrows indicate some representative double-positive cells. Scale bar, 50 μm. DAPI counterstain appears blue in all images. **B** The percentage of P21^+^ F4/80^+^ cells in senescent cells at the defect edge from PBS, D/Q, 6-OHDA and 6-OHDA + D/Q treatment mice. **C** UMAP plots showing the distribution of macrophage subpopulations in the PBS group and 6-OHDA group. **D** Bar plot illustrating the distribution of macrophage subpopulations across different treatment conditions after sub-clustering. **E** Expression levels of *Arg1* in macrophage subpopulations. **F** Aging signature scores of macrophage subpopulations. **G** Remodeling signature scores of macrophage subpopulations. **H** GO enrichment analysis of secretory proteins highly expressed in the (H) Arg1_1 and (I) Arg1_2macrophage subpopulation. **I** Ligand–receptor pairs involved in the interaction between Arg1_2 macrophages, *Emcn*⁺ *Pecam1*⁺ endothelial cells, and the suture.

Immunofluorescence staining showed a significant increase in P21^+^F4/80^+^ senescent macrophages in the 6-OHDA group, which was attenuated by Dasatinib and Quercetin (D/Q) treatment (Fig. 5A, B). This suggests that sympathetic nerve inhibition promotes the accumulation of senescent-like macrophages, contributing to defect repair. To identify responsive macrophage subsets, we performed secondary clustering and identified six subpopulations (Fig. 5C). Among these, clusters a and c were enriched at early time points and showed opposite trends between PBS- and 6-OHDA-treated mice at day 7 post-defect (Fig. 5C, D). Marker analysis revealed high Arg1 expression in both clusters, with cluster a expressing higher levels, designated as Arg1_1(a) and Arg1_2(c), respectively (Fig. 5E). Trajectory analysis showed that in PBS-treated mice, cells differentiated from b into Arg1_1, whereas in 6-OHDA-treated mice, cluster b differentiated into Arg1_2 (Supplementary Fig. S4A).

Importantly, the Arg1_2 subpopulation exhibited the highest enrichment of senescence gene signatures (Fig. 5F), suggesting its senescent-like phenotype. In addition, the Arg1_2 subpopulation exhibited the highest remodeling score (Fig. 5G). GO analysis indicated that Arg1_1 and Arg1_2 cells were both associated with vascular development and wound healing, while Arg1_2 cells were additionally enriched in ossification pathways (Fig. 5H, Supplementary Fig. S4B, C), suggesting a potential role in promoting osteogenesis. Cell-cell communication analysis revealed that Arg1_2 macrophages strongly interacted with endothelial cells through Spp1-Itga5/Itgb1 and Vegfa-Vegfr1/2 signaling—pathways known to promote angiogenesis (Fig. 5I). In interactions with suture cells, the most prominent signals were Spp1-Itgav/Itgb5, Spp1-Itgav/Itgb1, and Spp1-Cd44, all of which have been shown to enhance osteogenic differentiation.

Taken together, these results suggest that sympathetic nerve inhibition promotes calvarial defect repair by inducing a senescent-like Arg1_2 macrophage subpopulation, which facilitates angiogenesis and enhances osteogenesis within the suture, thereby promoting its regenerative potential.

## Discussion

In this study, we demonstrate that pharmacological inhibition of the peripheral sympathetic nervous system (SNS) significantly enhances calvarial bone repair through coordinated promotion of osteogenesis, angiogenesis, and modulation of immune cell functionality-specifically, the dynamic regulation of macrophage subpopulations.

This is consistent with previous studies, which have demonstrated the critical role of immune and stromal cell reprogramming in tissue regeneration [30]. Moreover, the role of nerve in-growth in tissue repair has gained increasing attention. Ectopic sympathetic nerve sprouting has been implicated in impairing bone healing by promoting bone resorption and inhibiting osteogenesis, as observed in previous studies on long bones [31]. Similarly, our data show that sympathetic nerve inhibition using 6-OHDA markedly enhances bone defect repair, as evidenced by increased bone volume and improved structural parameters on micro-CT analysis.

The calvarial sutures serve as a unique niche for stem cells involved in bone repair [20, 32]. Through transcriptomic and functional analyses, our study delineates the suture cell subpopulation as a pivotal mediator of osteogenesis, particularly evident following sympathetic denervation, via the upregulation of osteogenic signaling cascades, including PI3K-Akt and ECM-receptor interactions [33, 34]. Concurrently, pharmacological or genetic sympathetic inhibition augments this osteogenic phenotype through the downregulation of *Capn6*, a negative regulator of bone formation.

Angiogenesis is a crucial process in bone repair, with endothelial cells serving as the central cell types involved in angiogenesis during bone repair [13, 35, 36]. The VEGF signaling pathway has been shown to enhance the osteogenic differentiation of progenitor cells [37]. In our study, we found that inhibition of sympathetic nerves led to an increase in H-type vessels and enhanced interactions between endothelial cells and suture cells. Immunofluorescence staining of CD31 and EMCN further confirmed the increased vascularization. These interactions and the associated secretion of angiogenic factors likely facilitated angiogenesis and created a vascular microenvironment conducive to osteogenesis following sympathetic nerve inhibition.

Macrophages play a pivotal role in coordinating tissue repair through their functional plasticity [38]. Our study reveals significant changes in macrophage subpopulations following sympathetic nerve inhibition, including a shift from Arg1_1 to Arg1_2 macrophages. *Arg1*^+^ macrophages have been previously reported to play an important role in tissue repair [38]. Unlike the Arg1_1 subset, which is primarily associated with vasculature development and endothelial proliferation, the Arg1_2 macrophages exhibit enhanced ossification-related functions, likely contributing to the accelerated bone repair observed in our model. These findings are consistent with recent reports indicating that macrophages can secrete osteogenic factors to promote bone regeneration [39, 40].

Interestingly, we also observed a significant increase in macrophage senescence following sympathetic nerve inhibition. Although cellular senescence is often regarded as a pathological process, recent studies have highlighted its beneficial role in tissue repair by promoting a secretory phenotype that enhances regeneration [41, 42]. Our findings align with these observations, after sympathetic nerve inhibition, the proportion of senescent cells at the calvarial defect site significantly increased. Moreover, the use of senescence inhibitors abolished the beneficial effects of sympathetic nerve inhibition on bone repair, underscoring the functional importance of senescence in this context. Following sympathetic denervation, the number of senescent cells increased, and these senescent populations may contain components that suppress bone repair. However, treatment with 6-OHDA combined with DQ is insufficient to clearly distinguish between different types of senescent cells. At the same time, inhibition of sympathetic nerves may also directly affect calvarial defect repair through other stromal pathways. Further studies are needed to elucidate the underlying mechanisms and define the specific subtypes of senescence involved.

Our findings suggest that targeting sympathetic nerve activity and senescence-related pathways may represent a novel and clinically actionable strategy for enhancing bone regeneration. Given the limited regenerative capacity of calvarial bone, especially in elderly patients or individuals with large bone defects [43, 44], sympathetic nerve inhibition may offer a complementary approach to current surgical or stem cell-based therapies. Moreover, the identification of a senescent-like macrophage population with osteogenic potential raises the possibility of cell-based therapies that selectively modulate macrophage phenotypes to promote repair. Pharmacological modulation of SNS activity—either systemically or locally—could be developed into a targeted therapy for patients with delayed or non-healing bone defects. However, clinical translation requires careful evaluation of the specificity, dosing, and potential off-target effects of sympathetic inhibitors. Future studies should focus on developing delivery systems, such as hydrogel-based local administration, to achieve targeted modulation with minimal systemic side effects.

Our findings suggest that modulating SNS activity represents a promising therapeutic strategy for enhancing bone repair. Given the involvement of sympathetic nerves in various physiological and pathological processes, further studies are warranted to explore the long-term effects and safety of sympathetic nerve inhibition. Additionally, our study raises intriguing questions regarding the interplay between senescence and bone repair, particularly in the context of macrophage plasticity. Understanding how to fine-tune senescence to maximize its regenerative benefits while minimizing potential adverse effects could provide new avenues for therapeutic development.

While our study provides comprehensive insights into the cellular and molecular mechanisms underlying SNS-mediated regulation of bone repair, several limitations should be acknowledged. First, the use of 6-OHDA to inhibit SNS activity may have off-target effects on other peripheral nerves. Future studies employing genetic models or selective inhibitors could provide more specific insights. Second, our findings raise the possibility that senescent macrophages may contribute to H-type vessel formation. Sympathetic nerve inhibition induces senescent macrophages that secrete osteogenic and angiogenic factors, suggesting a potential mechanism by which neuroregulation could enhance type H vessel-mediated bone regeneration. Future studies are needed to clarify whether the effects of sympathetic suppression on H-type vessels and bone formation are directly mediated by these senescent macrophages. Third, our findings are based on murine models, which may not fully recapitulate human bone repair. Validation in human systems or larger animal models will be essential for translating these findings into clinical applications.

In conclusion, our study highlights the multifaceted roles of sympathetic nerves in regulating bone repair and identifies novel cellular and molecular mechanisms that can be targeted for therapeutic purposes. By integrating single-cell sequencing, functional analyses, and pharmacological interventions, we provide a foundation for understanding the dynamic interplay between the nervous system and bone regeneration. These findings offer novel insights for developing innovative strategies to enhance bone defect repair in clinical settings. Importantly, our findings offer new translational perspectives by demonstrating that pharmacological inhibition of sympathetic nerves and modulation of senescent macrophages may be leveraged to develop effective therapies for challenging clinical scenarios such as bone defects. Future work focusing on drug delivery strategies, biocompatibility, and regenerative efficacy in large animal models will be critical to bridge the gap from bench to bedside.

## Methods

### Animals

All animal experiments were conducted in accordance with the relevant guidelines and regulations, and approved by the Animal Care and Use Committee of Tongji University (Approval No. TJ-HB-LAC-2024-41). 10-week-old, male C57BL/6J mice were purchased from the SHANGHAI SLAC LABORATORY ANIMAL CO. LTD.

### Calvarial defect creation

For calvarial defect creation, anesthesia was performed with 2-3% isoflurane in 100% oxygen at a flow rate of 1 L/min and animals were operated upon on a warm animal surgery station according to previously published methods [14]. Firstly, hair overlying the calvaria was clipped, and a 4 mm skin incision was made over the right frontal bone. Next, a 1.8 mm diameter full thickness circular defect was created in the non-suture associated frontal bone using a micro surgical drill and a trephine drill bit (Xemax Surgical, Napa Valley, CA). Meticulous care was taken not to injure the underlying dura mater. Finally, the skin was sutured and the animal was monitored per established postoperative protocols. Post-operative monitoring was performed in accordance with institutional policy. Skulls were harvested up to 7d and 28d after defect, respectively.

### Intervention treatment

Two days before the calvarial defect surgery, the mice in the experimental group were injected subcutaneously with 6-Hydroxydopamine (6-OHDA; Sigma-Aldrich, H4381, 0.8 mg/80 µl per mouse) at the location of the right frontal bone on two consecutive days. The mice in the vehicle group were injected subcutaneously with vehicle solution (80 µl per mouse) at the location of the skull on two consecutive days. 6-OHDA was dissolved in vitamin C and phosphate buffer saline. For vehicle control group, vitamin C and phosphate buffer saline at the same concentration was performed.

For Dasatinib/Quercetin (D/Q) intervention in animals who were harvested on 28d, Dasatinib (5 mg/kg, MCE, HY-10181, USA) and Quercetin (25 mg/kg, MCE, HY-18085, USA) were administered orally. From the day of surgery as the first day of treatment, it continued for 14 days. Both compounds were dissolved in 4% DMSO and phosphate buffer saline.

### Senescent cell injection

To inhibit sympathetic nerve activity, mice were subcutaneously injected with 6-hydroxydopamine (6-OHDA; Sigma-Aldrich, H4381; 0.8 mg in 80 µl per mouse) at the right frontal bone site once daily for four consecutive days, beginning four days before calvarial defect surgery. Senescent cells were isolated on day 7 after calvarial injury and subsequently injected into the defect sites of mice that had received surgery three days prior. Mice were sacrificed and samples were collected on day 28 post-injury.

### Micro-CT

Samples were fixed in 4% paraformaldehyde solution for a full day and then scanned with a high-resolution micro-computed tomography imaging device (SkyScan 1294; Bruker MicroCT N.V, Kontich, Belgium). The scanning process adopted an image resolution of 10 mm, along with these particular settings: a 1 mm aluminum filter, an X-ray voltage set at 65 kVP, an anode current of 153 µA, an exposure duration of 65 ms, a frame averaging factor of 4, and a rotation increment of 0.3 degrees. Subsequently, three-dimensional renderings were reconstructed from the two-dimensional X-ray projections by leveraging the Feldkamp algorithm via a commercial software suite known as NRecon software (2.0.4.0 SkyScan, Bruker). In terms of conducting 3D morphometric evaluations of the images, CTVox and CTAn software programs were employed (1.13 SkyScan, Bruker). For the examination of calvarial defects, a cylindrical region of focus centered on each defect location was demarcated, with a diameter of 1.8 mm, a height of 1 mm, and a threshold value of 80. The extent of bone formation was analyzed and numerically quantified. After that, the ratio of bone volume to total volume (BV/TV) as well as the area of the bone defect was computed and measured precisely.

### Isolation and culture of mouse calvarial suture cells

Calvarial suture cells were isolated from wild-type C57BL/6J mice. After careful removal of the periosteum and dura mater, sagittal and coronal sutures were dissected together with ~0.5 mm of adjacent parietal and frontal bone on either side. The collected tissues were finely chopped and plated in 10-cm culture dishes. Cells were cultured in α-MEM (Gibco) containing 15% fetal bovine serum (FBS, Gibco), 100 U/mL penicillin, and 100 μg/mL streptomycin under standard conditions (5% CO₂, 37 °C). Within 3 to 5 days, suture-derived cells began to migrate out from the explants. The culture medium was refreshed every 3 days. Cells were passaged upon reaching confluency, and those at passage 2 or 3 were used for subsequent experiments.

Suture mesenchymal cells were isolated from wild-type C57BL/6J mice. After euthanasia, the sagittal and coronal sutures were carefully microdissected under a stereomicroscope. The excised tissues were minced into small fragments and placed in 10-cm culture dishes to allow cell outgrowth. After 3–4 days of culture under standard conditions (37 °C, 5% CO_2_), the migratory suture cells were collected and subjected to fluorescence-activated cell sorting (FACS) to enrich for mesenchymal populations. Cells were labeled with APC-conjugated antibodies against CD31, CD45, and Ter119 to exclude endothelial, hematopoietic, and erythroid lineage cells, respectively.

### siRNA knockdown

Purified mesenchymal cells were seeded in 6-well plates at an initial density of 2 ×105 cells/mL and cultured for 24 h prior to transfection. siRNA targeting *Capn6* and a scrambled control siRNA were purchased from Thermo Fisher Scientific. Transfections were carried out using TransIT-LT1 reagent (Mirus Bio, Madison, WI) following the manufacturer’s guidelines. Briefly, the transfection mix was prepared in 250 μL Opti-MEM (Gibco) containing 2.5 μg siRNA and 7.5 μL TransIT-LT1, incubated at room temperature for complex formation, and then added to the culture. Cells were maintained for an additional 48 h post-transfection. Knockdown efficiency was validated by quantitative real-time PCR (qPCR). Experiments were performed in triplicate (*n* = 3). Following Capn6 knockdown, cells were induced to undergo osteogenic differentiation by culturing in osteogenic induction medium (α-MEM containing 10% FBS, 50 μg/mL ascorbic acid, 10 mM β-glycerophosphate, and 100 nM dexamethasone) for 14 days. Osteogenic capacity was assessed by Alizarin Red S staining.

### Droplet-based scRNA-seq using the 10x Genomics Chromium platform

The frontal and parietal bones, which encompassed the sagittal and coronal suture and might or might not have a 1.8 mm defect (as shown in Supplementary Fig. S4A), were meticulously micro-dissected. Subsequently, they were digested with a mixture of collagenase Type I/II (1 mg/mL, Worthington Biochemical Corporation, Lakewood, NJ; LS004197 and LS004177) and Dispase II (2 mg/mL) for three rounds of 15 min each. Three animals were included in each group: those with intact bone, and those at 7 days and 28 days post-bone defect. The cell fractions obtained were then gathered and resuspended in red blood cell lysis buffer, left at room temperature for 10 min. After that, the digested samples were filtered through 40 μm sterile strainers. Next, the cells were washed with PBS and resuspended in 0.1% BSA in HBSS (Gibco, Island, NY). Cell viability was evaluated using Trypan blue, and it was found to be over 85%. Later, the cells were transported to the JHMI Transcriptomics and Deep Sequencing Core. The entire batch of cells was loaded onto the 10× Genomics chromium controller to fabricate single-cell barcoded droplets (GEMs) following the manufacturer’s guidelines for the 10× single-cell 3′ v2 chemistry, with the goal of having 10,000 cells per channel. The resultant libraries were sequenced on an Illumina NovaSeq S2 100 cycle device (manufactured in San Diego, CA). At the JHMI Transcriptomics and Deep Sequencing Core, CellRanger was employed to conduct sample demultiplexing, barcode processing, and single-cell gene counting (including Alignment, Barcoding and UMI Count).

For encapsulating single cells into droplet emulsions, a Chromium Single-Cell apparatus (from 10× Genomics) was utilized. To construct the scRNA-seq libraries, the protocol provided by 10× Genomics was adhered to, making use of the Chromium Single-Cell 3’ Gel Bead and Library V3 Kit. Each channel of the instrument was filled with isolated single cells. Reverse transcription and library preparation were executed using a Bio-Rad C1000 Touch thermal cycler equipped with a 96-deep well reaction module. A sum of 12 cycles was employed for cDNA amplification and the subsequent sample index PCR stage. The average fragment length of the resulting 10× cDNA libraries was measured using a fragment analyzer (from AATI) and quantified via PCR with the KapaL Library Quantification Kit designed for Illumina. Eventually, the libraries were sequenced using the NovaSeq 6000 Sequencing System.

### Preliminary processing of scRNA-seq raw data from 10x Genomics

Cell Ranger software (version 6.1.1) was used for the initial processing of single-cell sequencing data. The process consisted of aligning reads, performing estimation and filtering, and producing feature-barcode matrices. Pre-processing mouse reference genome (mm10) was downloaded form https://www.10xgenomics.com/cn/support/software/cell-ranger/latest/release-notes/cr-reference-release-notes#2020-a. Initially, raw reads were mapped to the mouse reference genome. Subsequently, the ‘count’ function with its default settings was then used to create feature-barcode matrices for further analysis.

### Cell clustering and annotation

Seurat (4.1.1) was used to perform downstream analysis. Cells were considered low-quality and excluded if they had fewer than 200 genes or if their mitochondrial gene ratio exceeded 15%. Normalization and scaling of each sample’s expression matrix were performed using the ‘SCTransform’ function, after which ‘PrepSCTIntegration’ and ‘FindIntegrationAnchors’ functions were employed to identify features and anchors for further integration. The ‘IntegrateData’ function was employed to combine all datasets. Subsequently, the ‘ScaleData’ and ‘RunPCA’ functions were utilized for scaling and performing principal component analysis (PCA) on the integrated datasets.

Subsequently, the datasets were clustered into multiple groups using the ‘FindNeighbors’ and ‘FindClusters’ functions for unsupervised data analysis. Dimensionality reduction on the datasets was performed with the ‘RunUmap’ function. To examine the specificity of gene expression in each cluster, the ‘FindAllMarkers’ function (avg_log2FC > 0.25, p_val_adj < 0.05) was employed to pinpoint genes that are highly expressed in specific clusters and cell types based on marker gene expression.

### Cell–cell communication analysis

CellChat [45] (v.1.4.0, R package) was used to assess the interactions between various cell types. CellChat uses gene expression data provided by the user to estimate the likelihood of cell–cell communication by combining gene expression with a pre-existing database of known interactions among signaling ligands, receptors, and their cofactors. This study examined cell–cell interactions separately under various conditions using the standard procedure. A CellChat object was created using normalized count data from each condition, and the suggested preprocessing functions were applied to analyze individual datasets with default settings. For inferring cell-cell communication, the CellChatDB.mouse database served as the source, using all types of ligand-receptor interactions. Any communications with fewer than 10 cells were excluded from the analysis.

### Gene set preparations

The aging-related gene set was derived from the previously published SenMayo gene set [46]. The gene sets for lysosome, ossification, tissue remodeling, proliferation and migration were obtained from the Gene Ontology (GO) database by selecting genes annotated as related to lysosome, ossification, tissue remodeling, stem cell proliferation and cell migration (Supplementary Table S3). Then gene set scores were calculated by the ‘AddModuleScore’ function of Seurat.

### Analysis of defect responsiveness using Augur

To evaluate the responsiveness of cell types to Sympathetic inhibition, Augur scores were computed for PBS and 6-OHDA treated samples. This was achieved by utilizing the calculate_auc function from the R package Augur [47] (version 1.0.3). The Seurat object, annotated with ‘cell type’ and ‘time point’ labels, was provided as input for the analysis.

### Gene Ontology (GO) and KEGG enrichment analysis

The R package clusterProfiler was utilized to conduct GO and KEGG enrichment analyses on marker genes from different cell clusters, differentially expressed genes (DEGs) between PBS- and 6-OHDA-treated samples, as well as DEGs across various time points. ggplot2 (version3.3.6) were used to visualize the enrichment results.

### Histology and immunohistochemistry

After radiographic imaging, samples were transferred to 14% EDTA for decalcification for 14–21 days. Samples were then embedded in optimal cutting temperature compound (OCT) and sectioned in a coronal plane at 15 or 50 mm thickness. For immunofluorescent staining, sections were incubated with the following primary antibodies: anti-CD31 (1:100, Abcam 28364, USA), anti-EMCN (1:200, Santa Cruz sc-65495, USA), anti-TH (1:200, Abcam 137869, USA), and anti-Tubb3 (1:200, Abcam 18207). Sections were washed with phosphate buffered saline (PBS) three times, 10 min each. All sections were blocked with 5% goat serum in PBS for 45 min at RT. Primary antibodies were added to each section at their respective dilutions and incubated at 4 °C overnight. Next, the DyLight Fluor 594 goat anti-rabbit IgG (H + L) polyclonal (1:200, Abcam, 150084, USA) or Alexa Fluor 488 goat anti-rat IgG (1:200, Cell Signaling Technology, 4416S, USA) was used as the secondary antibody. Sections were counterstained with DAPI mounting medium (H-1800, Vector laboratories, USA). All histological sections were examined under a LEICA confocal microscope (LEICA, German).

For HE staining, the slices were stained in hematoxylin solution (3971, Sigma, USA) for about 3 min and rinsed in running water to develop the blue-purple color of the nuclei. Then, the slices were stained in eosin solution (G1100, Solarbio, China) for 1 min and rinsed again. Nest, the slices were dehydrated through an alcohol series (70%, 90%, and 100% ethanol) and cleared in xylene. Finally, the neutral gum was applied and a coverslip was placed over the section for microscopic examination.

For SA-β-gal staining, strictly follow the instructions (Damas, C8262, China). Briefly, use the β-galactosidase staining fixative solution to fix the sample for 20 min at room temperature. Then, after washing with PBS for 15 min, use the β-galactosidase staining working solution and incubate it at 37 °C for 12 h. Next, stain with Nuclear Fast Red (C0151, Beyotime, China) for 5 min at room temperature. Finally, after dehydrating with absolute ethanol and clearing with xylene, mount the sample with neutral balsam.

### Flow cytometry

First, extract the calvarial cells around the calvarial defect of mice 7 days after calvarial defect surgery. Use type I (1 mg/mL, 17100-017, Gibco, USA) and type II collagenases (1 mg/mL, 17101-015, Gibco, USA) to dissociate the cells into single cells. Then, add flow cytometry antibodies (including APC-Cy™7 Rat Anti-Mouse CD45 (BD Pharmingen™, 557659, USA), APC Rat Anti-Mouse F4/80 (BD Pharmingen™, 566787, USA), PE Rat Anti-CD11b (BD Pharmingen™, 553311, USA), APC Rat Anti-Mouse Ly-6G (BD Pharmingen™, 560599, USA), APC Rat Anti-Mouse PDGFRα (BD Pharmingen™, 562777, USA)) and incubate them at 4 °C for 20 min. As for SA-β-gal staining, follow the instructions exactly (CellEvent™ Senescence Green Flow Cytometry Assay Kit, C10840). Briefly, stain cell surface antigens with above antibodies. Then, resuspend the cells in Fixation Solution for 10 min. Next, resuspend the cells in Working Solution for 1 h in 37 °C protected from light. Finally, after thorough washing, resuspend the cells with the resuspension solution and transfer them into flow cytometry tubes for analysis on the instrument.

### Isolation of senescent macrophages in vivo

To isolate senescent macrophages from mouse tissue, single-cell suspensions were prepared from calvarial bone. senescence-associated marker using the Cell Meter™ Cellular Senescence Activity Assay Kit (Red Fluorescence, AAT Bioquest) according to the manufacturer’s instructions. Senescent macrophages were then sorted by fluorescence-activated cell sorting (FACS) and collected for downstream analyses.

### Isolation of *Arg1*^+^ macrophages in vivo

To isolate *Arg1*⁺ macrophages from mouse tissue, single-cell suspensions were prepared from calvarial bone of PBS- or 6-OHDA-treated mice. Cells were stained with antibodies against CD45 (BD Pharmingen™, 557659, USA), F4/80 (BD Pharmingen™, 566787, USA), and Arg1 (invitrogen, 17-3697-82, USA) according to the manufacturer’s instructions. CD45⁺F4/80⁺Arg1⁺ macrophages were then sorted by FACS and collected for downstream analyses.

### Statistical analysis

Quantitative data are presented as mean ± SD. Statistical analysis was conducted using Graphpad software (RRID:SCR_002865). All data followed a normal distribution. For comparing two groups, Student’s *t* test was applied, while a one-way ANOVA test was utilized for comparing three groups. Significance was determined at *p < 0.05, **p < 0.01, ***p < 0.001 and ****p < 0.0001.

## Supplementary information

Supplementary materials

Supplementary materials Table S1

Supplementary materials Table S2

Supplementary materials Table S3

## Data Availability

The raw single-cell RNA sequencing data reported in this study have been deposited in the Gene Expression Omnibus (GEO) under accession number GSE298251.
