# Supplementary material for: Sympathetic nerve inhibition enhances calvarial bone repair via senescent macrophage-induced osteogenesis and angiogenesis
Source: Cell Death Discov. 2025 Dec 10;11:564. doi: 10.1038/s41420-025-02886-y (PMC12728170; doi:10.1038/s41420-025-02886-y)
Supplement: Supplementary file 1 — Supplementary materials [file 41420_2025_2886_MOESM1_ESM.docx]

**Supplementary materials**


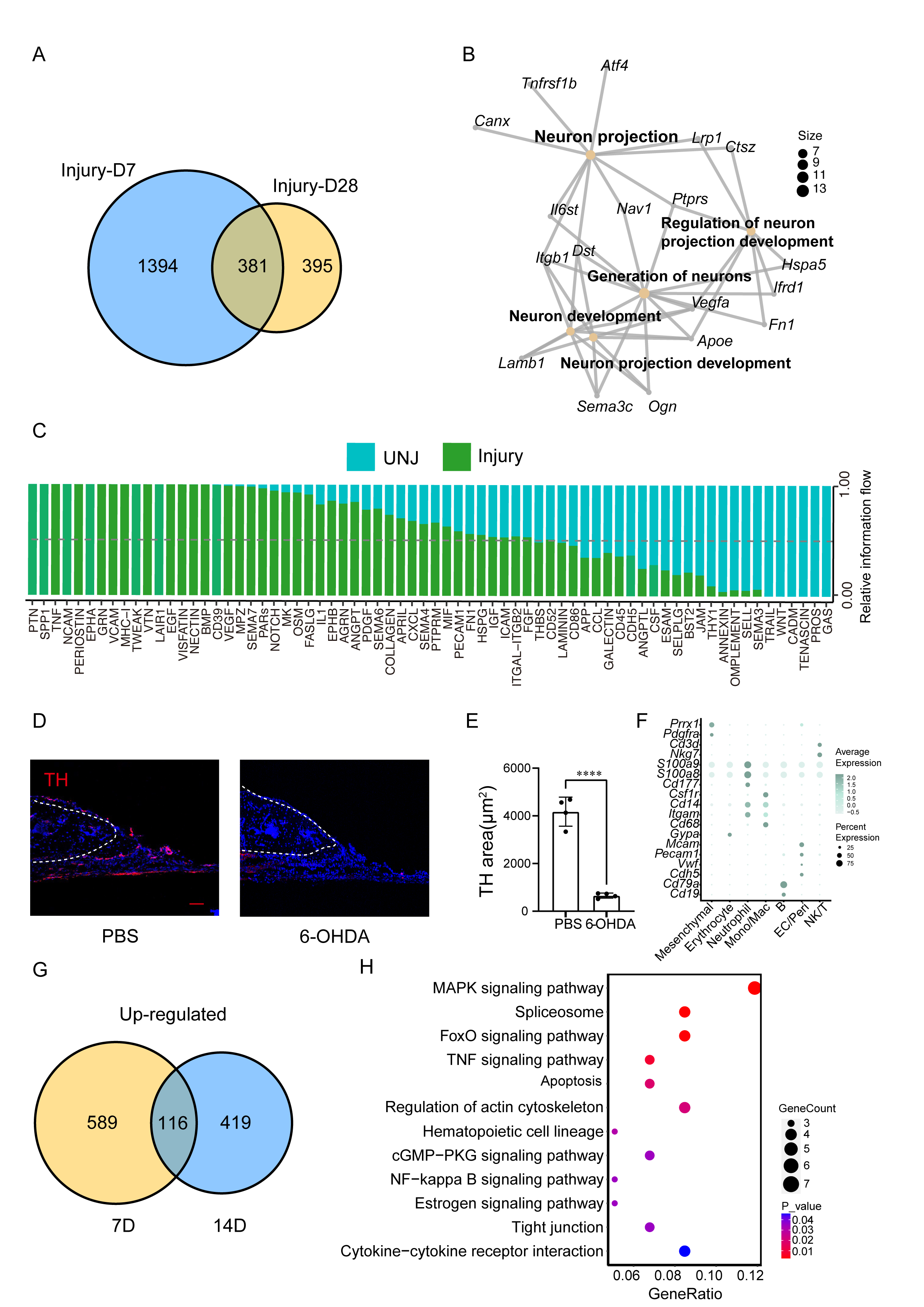


**Supplement Figure S1.** **Changes in Cellular communication after cavarial bone injury**

**A**. Venn diagram showing the overlap of genes upregulated at 7 and 28 days after injury.

**B**. GO enrichment analysis of secretory proteins consistently upregulated after defect.

**C**. **Signaling pathways involved in cell–cell communication that are significantly altered between uninjured and injured mice.**

**D**. TH staining results of sympathetic nerves in the PBS-treated and 6-OHDA-treated groups.

**E**. Statistical analysis of TH area in the PBS-treated and 6-OHDA-treated groups.

**F**. Dot plots showing marker gene expression across various cell types.

**G.** Venn diagram showing the overlap of genes upregulated at days 7 and 14 in the 6-OHDA-treated group compared to the PBS group.

**H**. Bubble plot showing KEGG pathway enrichment of genes significantly upregulated in both day 7 and day 14 samples following 6-OHDA treatment.


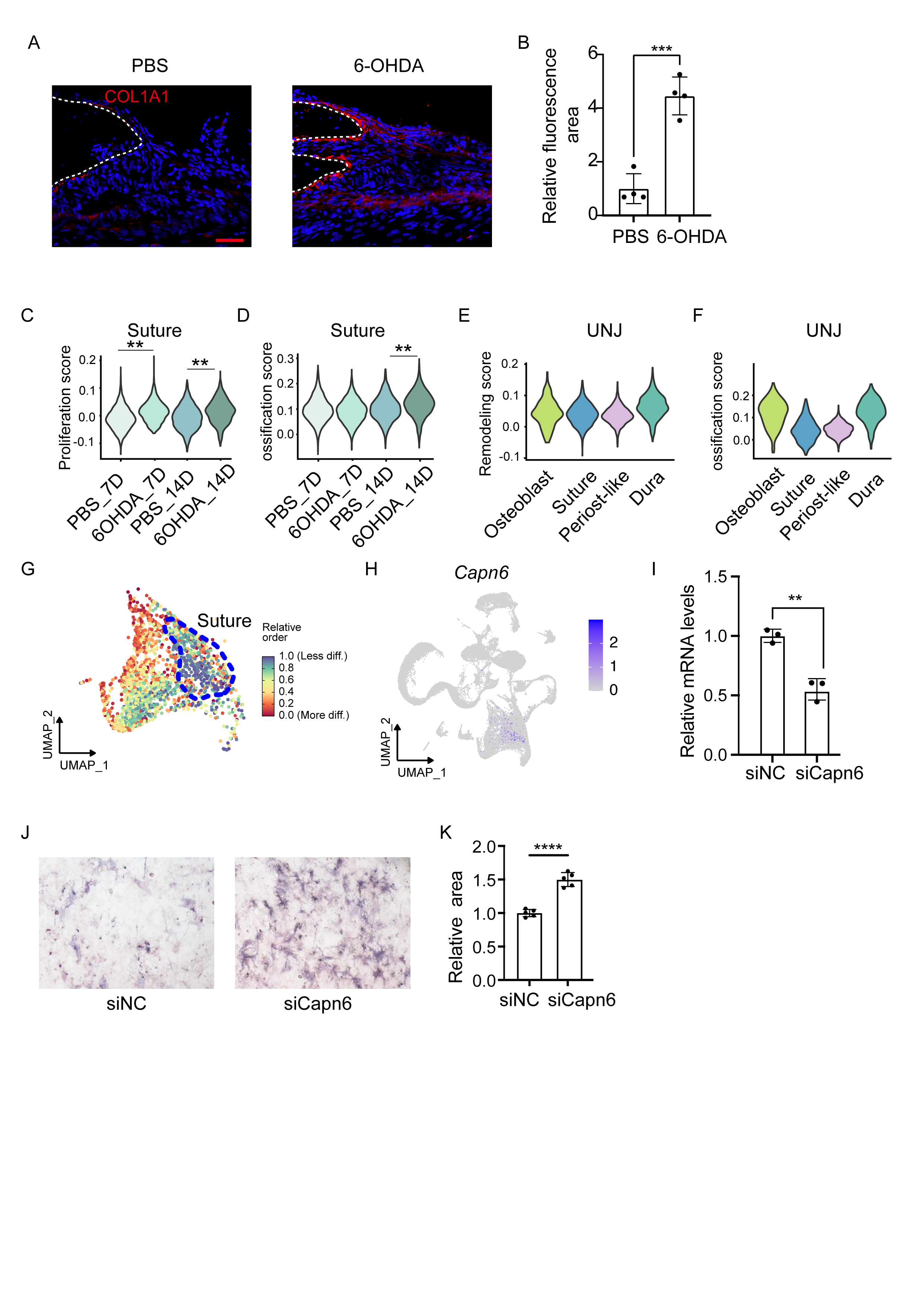
**Supplement Figure S2. Inhibition of sympathetic nerves enhances the osteogenic function of the suture.**

1. Immunohistochemical staining of COL1A1 at the defect edge from PBS or 6-OHDA treatment mice.

**B.** Quantification of COL1A1 immunoreactivity within the calvarial defect. Dashed white lines indicate bone edge. Scale bar, 100 μm. DAPI counterstain appears blue in all images. In graphs, each dot represents a single animal; n=4 per group. Data are represented as mean ± SD. ***p < 0.01.

**C-D**. (C) Proliferation score and (D) ossification score of the suture under different treatment conditions.

**E-F**. (E) Remodeling score and (F) ossification score of different mesenchymal cell subpopulations under normal conditions.

**G**. CytoTRACE2 plot showing cellular stemness across different cell subpopulations.

**H**. UMAP plot showing the expression of *Capn6* across all cell populations.

**I.** Knockdown efficiency of siCapn6. Relative mRNA expression of Capn6 was measured by qRT-PCR in cells transfected with siCapn6 compared with negative control siRNA (siNC). Data are presented as mean ± SEM (n = X). **p < 0.01.

**J-K.** ALP staining and quantification. ALP staining was performed on cells after 14 days of osteogenic induction. (J) Representative images of each group are shown , and (K) the ALP-positive area was quantified using ImageJ. Data are presented as mean ± SEM (n = X). ****p < 0.0001.


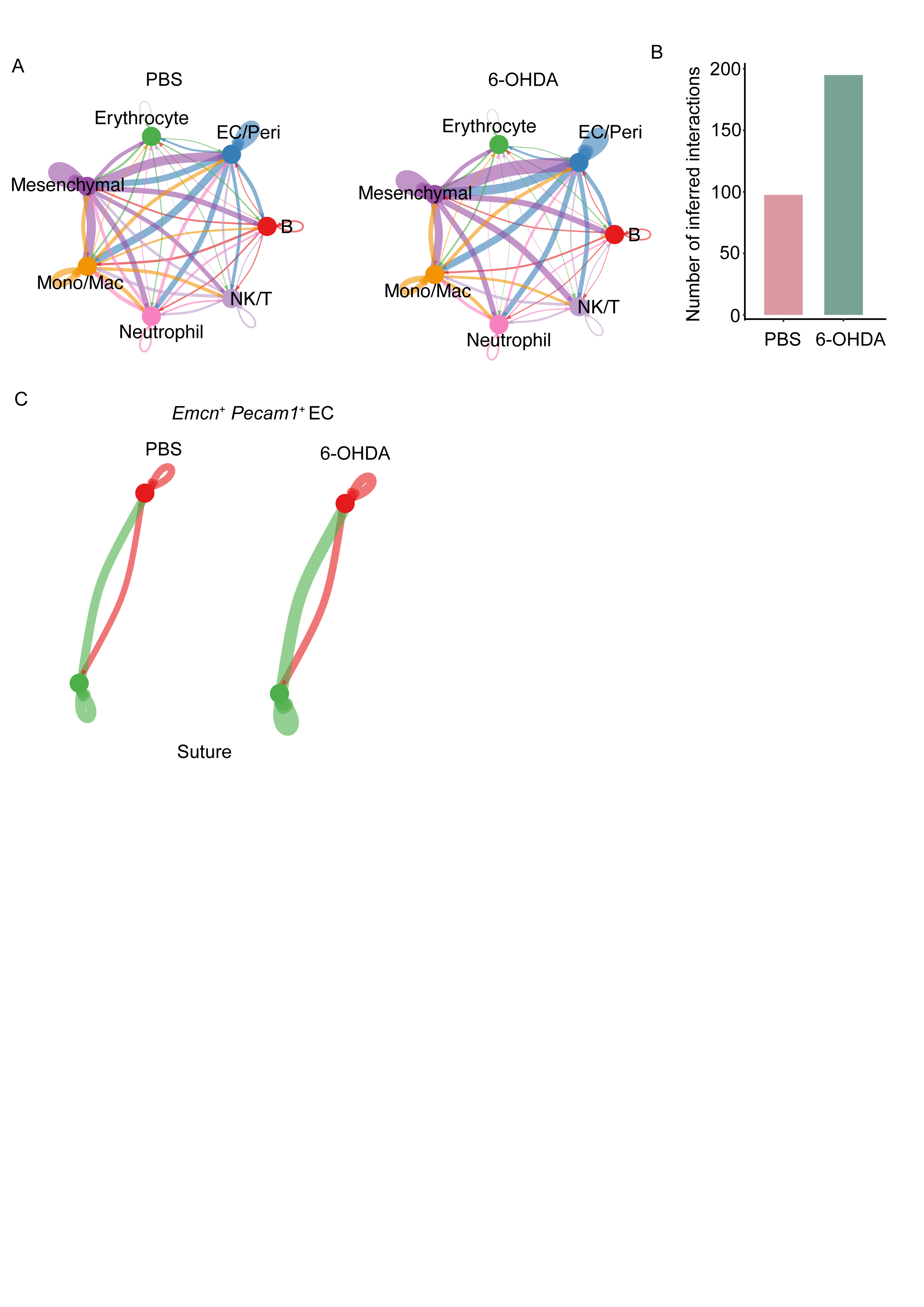


**Supplement Figure S3. Inhibition of sympathetic nerves promotes endothelial cells-mesenchymal cells interactions**

**A.** Alterations in cell–cell communication among distinct cell subpopulations between 6-OHDA- and PBS-treated groups**.**

**B.** Comparison of the number of cell–cell communication events between PBS and 6-OHDA treatment groups.

**C**. The number of interactions between *Emcn*^+^ *Pecam1*^+^ endothelial cells and suture in the 6-OHDA- and PBS-treated groups.


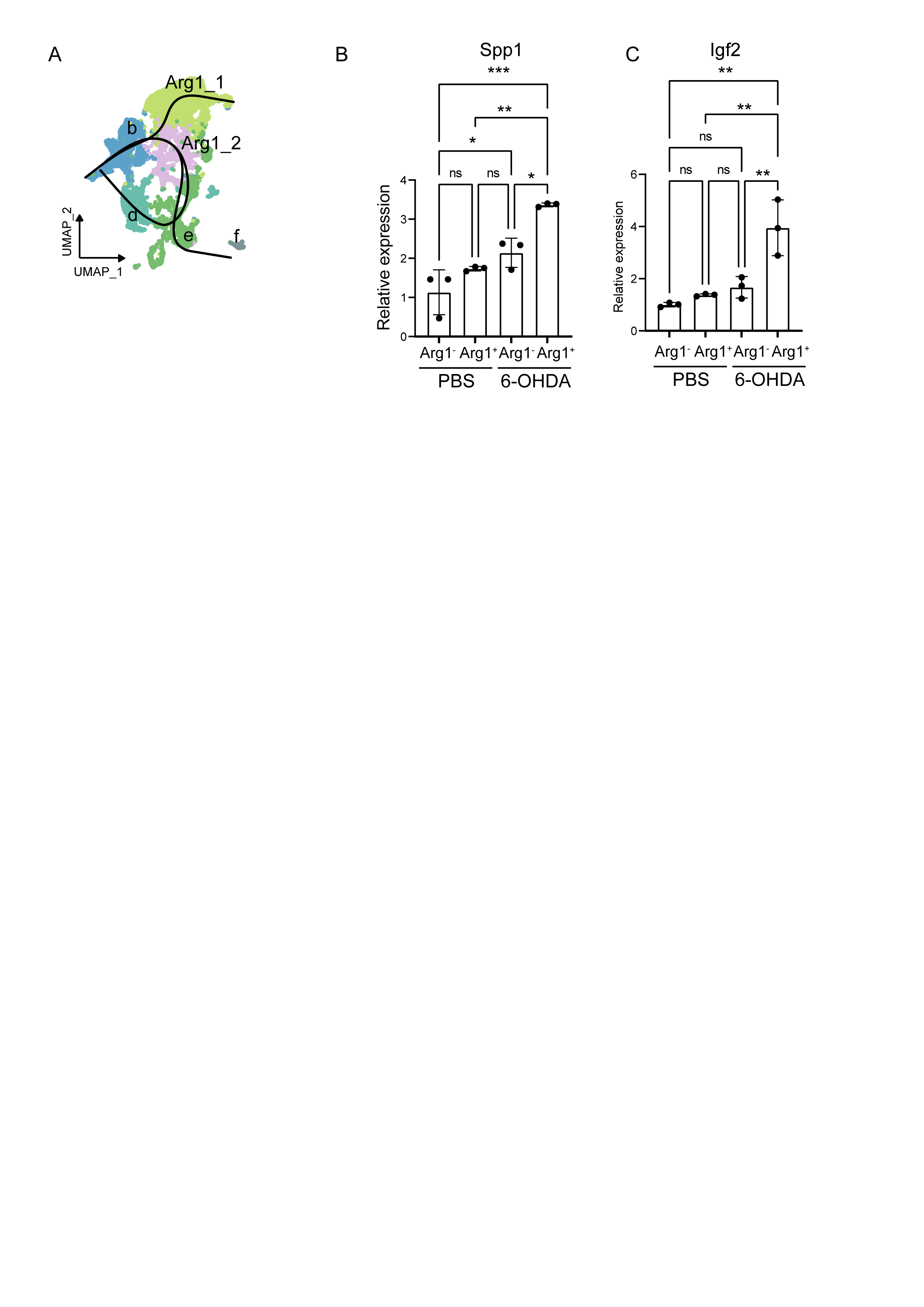


**Supplement Figure S4. Inhibition of sympathetic nerves facilitates the generation of novel macrophage subpopulations**

**A.** CytoTRACE2 plot showing the differentiation trajectory of macrophage subpopulations. **B-C.** Quantification of representative ossification pathway genes Spp1 (B) and Igf2 (C) by qRT-PCR in Arg1⁺ and Arg1⁻ cells isolated from PBS- and 6-OHDA–treated groups. Data are presented as mean ± SEM (n = X). *p < 0.05, **p < 0.01, **p < 0.001versus PBS control.

**Table S1**. Overlap of genes upregulated at 7 and 28 days after injury.

**Table S2**. Intersection of genes significantly up-regulated in both day 7 and day 14 samples after 6-OHDA treatment.

**Table S3**. Gene signatures of ossification, aging, tissue remodeling, proliferation and migration.
